# Design, synthesis, anticancer evaluation and molecular docking studies of 1,2,3-triazole incorporated 1,3,4-oxadiazole-Triazine derivatives

**DOI:** 10.1016/j.heliyon.2023.e15935

**Published:** 2023-05-03

**Authors:** Sujana Oggu, Parameswari Akshinthala, Naresh Kumar Katari, Laxmi Kumari Nagarapu, Srimannarayana Malempati, Rambabu Gundla, Sreekantha Babu Jonnalagadda

**Affiliations:** a Department of Chemistry, GITAM School of Science, GITAM (Deemed to be University), Hyderabad, Telangana, 502 329, India; b G. Narayanamma Institute of Technology & Science, Hyderabad, Telangana, 500 104, India; c Department of Science and Humanities, MLR Institute of Technology, Dundigal, Medchal, Hyderabad, Rudraram, 500043, India; d School of Chemistry & Physics, College of Agriculture, Engineering & Science, Westville Campus, University of KwaZulu-Natal, P Bag X 54001, Durban, 4000, South Africa

**Keywords:** Altretamine, 1,3,5-Triazine, 1,2,3-Triazole, Mubritinib, Anticancer activity

## Abstract

A new library of 1,2,3-triazole-incorporated 1,3,4-oxadiazole-triazine derivatives (9a-j) was designed, synthesized, and tested in vitro for anticancer activity against PC3 and DU-145 (prostate cancer), A549 (lung cancer), and MCF-7 (breast cancer) cancer cell lines using the MTT assay with etoposide as the control drug. The compounds exhibited remarkable anticancer activity, with IC50 values ranging from 0.16 ± 0.083 μM to 11.8 ± 7.46 μM, whereas the positive control ranged from 1.97 0.45 μM to 3.08 0.135 μM. Compound 9 d with a 4-pyridyl moiety shown exceptional anticancer activity against PC3, A549, MCF-7, and DU-145 cell lines, with IC50 values of 0.17 ± 0.063 μM, 0.19 ± 0.075 μM, 0.51 ± 0.083 μM, and 0.16 ± 0.083 μM, respectively.

## Graphical abstract

**Figure:**
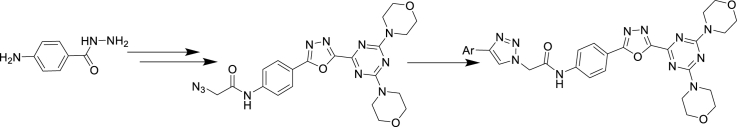

## 1 Introduction

**Fig. 1:**
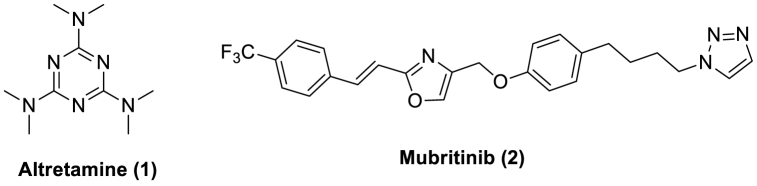
Structures of altretamine (1) and Mubritinib (2).

In medicinal chemistry, nitrogen heteroatom-containing heterocyclic scaffolds serve as active pharmaceutical components in the design and development of new chemotherapeutics. Apart from 1,3,5-triazines, nitrogen atoms with six-membered heterocyclic motifs are among the most recognized nitrogen atoms with a particular role in therapeutics. These triazine compounds exhibited various pharmacological activities, including anti-inflammatory [1], anticancer [2,3], anti-Alzheimer's [4], antibacterial [5], antiviral [6], and antifungal [7]. The U.S Food and Drug Administration has sanctioned the therapeutic agent, namely altretamine (1, Fig. 1), which bears 1,3,5-triazine moiety as a main backbone of the drug structure and is employed for the therapeutics of various cancers [[8], [9], [10]].

Similarly, 1,2,3-triazoles are nitrogen-containing five-membered heterocyclic aromatic scaffolds and contribute significantly to the medicinal field. They demonstrated various biological activities like anticancer [11], antimalarial [12], antitubercular [13], *anti*-HIV [14], antifungal [15], antibacterial [16], antidiabetic [17,18], and antineoplastic [[19], [20], [21]]. Generally, these 1,2,3-triazoles are prepared through a “Click” reaction involving copper-catalyzed (CuAAC) cyclo-addition reaction between various terminal alkynes and azides to give 1,2,3-triazoles. This method was first developed by Sharpless et al. in the year 2002 [[21], [22], [23], [24], [25], [26]]. Among various types of 1,2,3-triazoles, one of the molecules, namely Mubritinib (**2**) is, displayed anticancer activity by inhibition of protein tyrosine kinase, and it completed phase-I clinical trials [27].

We designed and prepared the unique library of 1,2,3-triazole linked 1,3,4-oxadiazole-triazine derivatives by monitoring the biological features and working actively (9a-j).

Furthermore, these compounds are examined for anticancer activity in vitro against four different cancer cell lines, including PC3 and DU-145 (prostate cancer), A549 (lung cancer), and MCF-7 (melanoma) (breast cancer).

## 2 Results and discussion

### 2.1 Chemistry

**Scheme 1:**
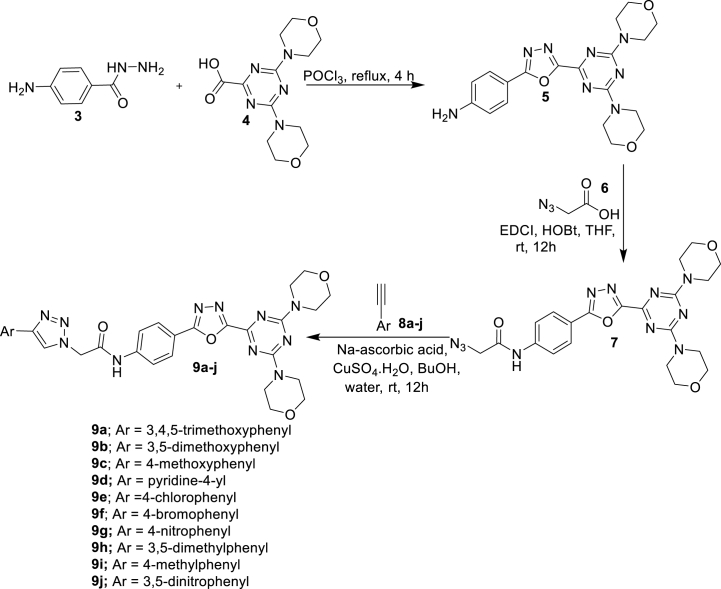
Synthesis of 1,2,3-Triazole incorporated 1,3,4-Oxadiazole-Triazine derivatives.

The new series of 1,2,3-triazole incorporated 1,3,4-oxadiazole-triazines (**9a-j**) was shown in Scheme 1. The intermediate 4-aminobenzohydrazide (**3**) underwent cyclization with 4,6-dimorpholino-1,3,5-triazine-2-carboxylic acid (**4**) in the presence of POCl_3_ then afforded the new 1,3,4-oxadiazole compound **5**. Further, this 1,3,4-oxadiazole was coupled with 2-azidoacetic acid (**6**) in the presence of EDCI and HOBt dissolved in dry THF at atmospheric temperature over 12 h time period then obtained compound 2-azido-N-(4-(5-(4,6-dimorpholino-1,3,5-triazin-2-yl)-1,3,4-oxadiazol-2-yl)phenyl)acetamide (**7**). This intermediate underwent 'Click reaction” by reacting with various terminal alkynes (**8a-j**) by utilizing Na-ascorbic acid and CuSO_4_·H_2_O in BuOH/water at an atmospheric temperature over 12 h time period then gave final target derivatives **9a-j.**

### 2.2 Biological evaluation - *in vitro* cytotoxicity

The synthesized ten different 1,2,3-triazole linked 1,3,4-oxadiazole-triazine derivatives (**9a-j**) were examined in vitro for their anticancer activity against four other cancer cell lines like PC3 & DU-145 (prostate cancer), A549 (lung cancer) and MCF-7 (breast cancer) by the utilizing of MTT assay with etoposide act as + ve control and the obtained results was incorporated in Table 1. These synthesized derivatives shown anticancer activities with IC_50_ values ranging from 0.16 ± 0.083 μM to 11.8 ± 7.46 μM, whereas the positive control ranged from 1.97 ± 0.45 μM to 3.08 ± 0.135 μM, respectively. Among them, the compounds **9a, 9b, 9d, 9g** and **9j** exhibited potent activity against particular cell lines. The SAR (Structure-activity relationship) studies of these derivatives determine that compound **9a** with 3,4,5-trimethoxyphenyl ring attached to 1,2,3-triazole moiety showed promising action against PC3, A549, MCF-7 and DU-145 cell lines with IC_50_ values of 0.56 ± 0.09 μM; 1.45 ± 0.74 μM; 1.14 ± 0.65 μM and 2.06 ± 0.92 μM. Whereas the compound **9b** with 3,5-dimethoxyphenyl ring attached to 1,2,3-triazole moiety exhibited decreased activity against PC3, A549, MCF-7 and DU-145 cell lines with IC_50_ values of 2.18 ± 1.93 μM, 1.90 ± 0.83 μM, 1.94 ± 0.89 μM, and 1.75 ± 0.78 μM than **9a**. The compound **9c** with 4-methoxyphenyl showed significantly lower activity against MCF-7 and DU-145 cell lines with IC_50_ values of 3.92 ± 2.14 μM and 5.64 ± 3.12 μM with **9a** & **9b**. The compound **9h** with 3,5-dimethylphenyl ring attached to 1,2,3-triazole moiety showed poor activities against PC3, A549, and MCF-7 cell lines with IC_50_ values of 4.13 ± 2.75 μM, 6.33 ± 4.84 μM, and 8.30 ± 5.35 μM. The compound **9i** with 4-methylphenyl ring attached to 1,2,3-triazole moiety also showed poor activities against PC3, A549, and MCF-7 cell lines with IC_50_ values of 7.21 ± 3.48 μM, 5.95 ± 4.26 μM, and 10.3 ± 5.42 μM. Interestingly, compound **9d** with 4-pyridyl moiety showed remarkable anticancer activity against PC3, A549, MCF-7, and DU-145 cell lines with IC_50_ values of 0.17 ± 0.063 μM, 0.19 ± 0.075 μM; 0.51 ± 0.083 μM and 0.16 ± 0.083 μM. The replacement of 4-pyridyl ring with 4-chlorophenyl resulted in compound **9e** displaying less anticancer activity against PC3, MCF-7 and DU-145 cell lines with IC_50_ values of 3.24 ± 2.51 μM, 3.74 ± 2.03 μM, and 6.13 ± 4.57 μM. As well as, the compound **9f** contained 4-bromophenyl substituent and exhibited inferior activity selectively on A549 cell line with IC_50_ value of 11.8 ± 7.46 μM. The compound **9g** with 4-nitrophenyl ring showed moderate activity against PC3, A549, MCF-7 and DU-145 cell lines with IC_50_ values of 2.32 ± 1.64 μM, 2.61 ± 1.93 μM, 2.94 ± 2.06 μM, and 2.12 ± 1.57 μM. Whereas, compound **9j** with 3,5-dinitrophenyl ring showed increased activity against PC3, A549, MCF-7 and DU-145 cell lines with IC_50_ values of 0.93 ± 0.086 μM, 1.24 ± 0.88 μM, 1.44 ± 0.93 μM, 1.65 ± 0.74 μM.

**Table 1:** *In vitro* cytotoxicity of newly synthesized derivatives **9a-j** with IC_50_ in μM.

Compound	PC3	A549	MCF-7	DU-145
9a	0.56 ± 0.09	1.45 ± 0.74	1.14 ± 0.65	2.06 ± 0.92
9b	2.18 ± 1.93	1.90 ± 0.83	1.94 ± 0.89	1.75 ± 0.78
9c	–	–	3.92 ± 2.14	5.64 ± 3.12
9d	0.17 ± 0.063	0.19 ± 0.075	0.51 ± 0.083	0.16 ± 0.083
9e	3.24 ± 2.51	–	3.74 ± 2.03	6.13 ± 4.57
9f	–	11.8 ± 7.46	–	–
9g	2.32 ± 1.64	2.61 ± 1.93	2.94 ± 2.06	2.12 ± 1.57
9h	4.13 ± 2.75	6.33 ± 4.84	8.30 ± 5.35	–
9i	7.21 ± 3.48	5.95 ± 4.26	10.3 ± 5.42	–
9j	0.93 ± 0.086	1.24 ± 0.88	1.44 ± 0.93	1.65 ± 0.74
Etoposide	2.39 ± 1.56	3.08 ± 0.135	2.11 ± 0.024	1.97 ± 0.45

“-” = No active.

### 2.3 Molecular docking studies

**Fig. 2:**
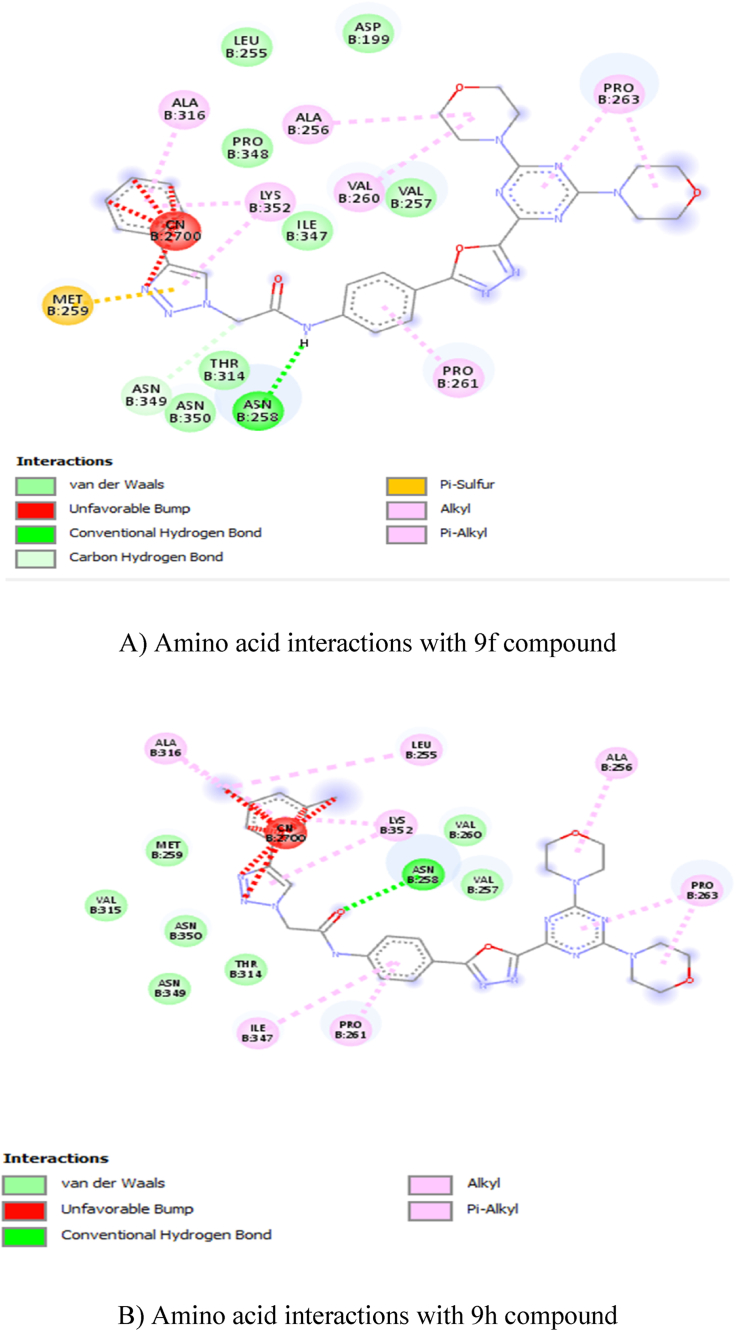

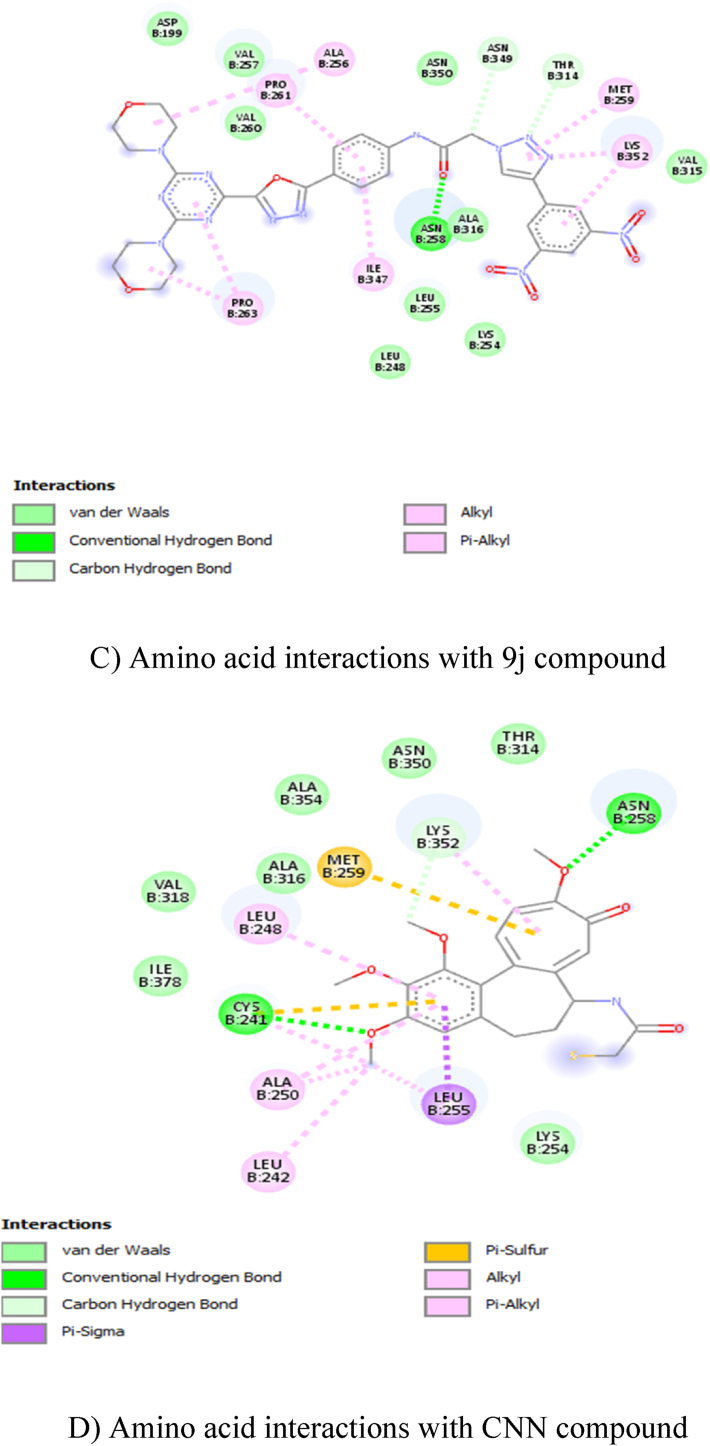
2D Ligand interaction diagrams for the top pose for docking A) 9f, B) 9h, C) 9j and D) CNN into the binding site colchicine of tubulin. A) Amino acid interactions with 9f compound B) Amino acid interactions with 9h compound C) Amino acid interactions with 9j compound D) Amino acid interactions with CNN compound

**Fig. 3:**
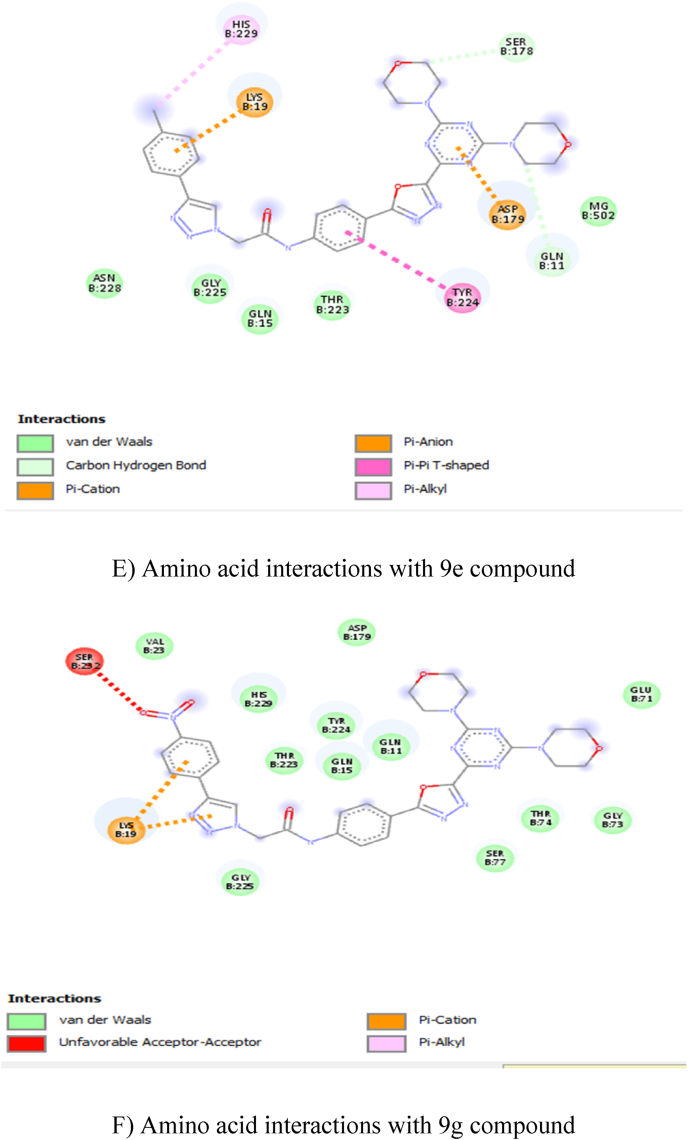

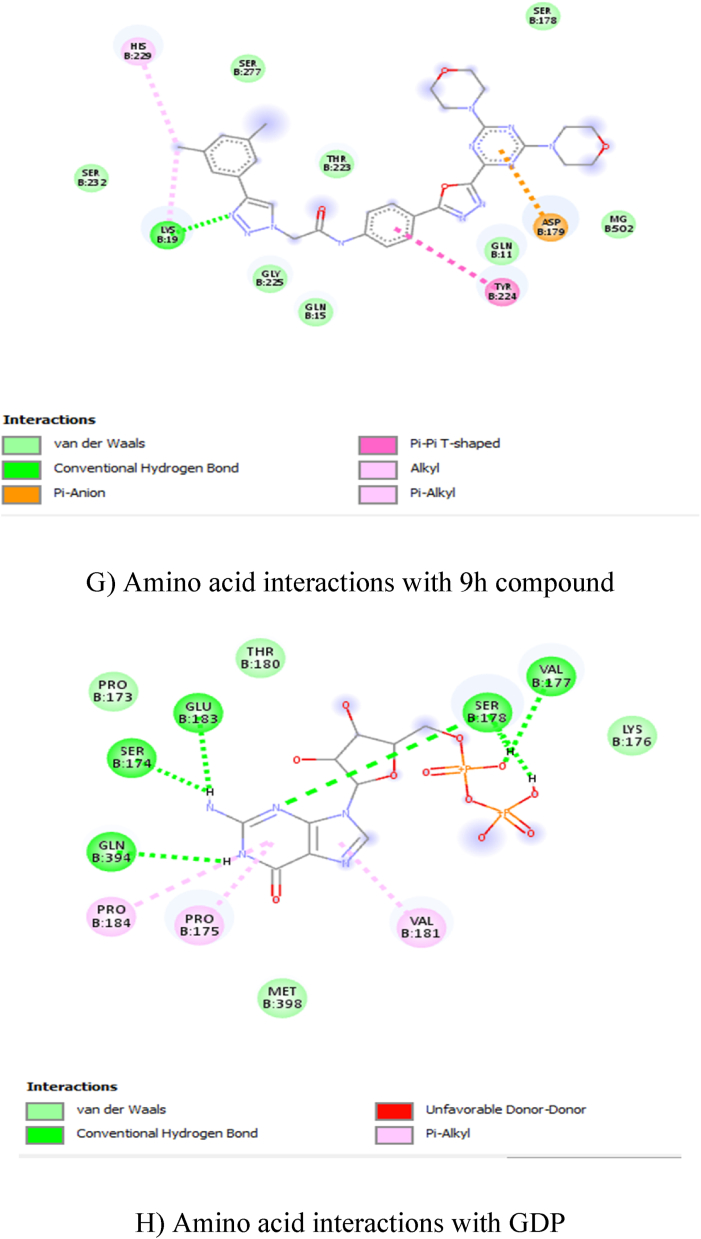
2D Ligand interaction diagrams for the top pose for docking 9e, 9g, 9h and GDP at the GDP binding site of tubulin. E) Amino acid interactions with 9e compound F) Amino acid interactions with 9g compound G) Amino acid interactions with 9h compound H) Amino a\cid interactions with GDP

In total ten compounds, **9h** (−9.6 kcal/mol), **9f** (−9.4 kcal/mol), **9j** (−9.4 kcal/mol), showed good activity in the tubulin CNN (colchicine) binding site (Fig. 2: A, B, C, and D), and **9e** (−8.2 kcal/mol), **9g** (−8.1 kcal/mol), **9h** (−8.3 kcal/mol) compounds showed good activity on tubulin GDP binding site (Fig. 3: E, F, G, and H). Nevertheless, none of the compounds showed good activity when compared to GDP (−9.4 kcal/mol) at GDP binding site (Table 2). The amino acid interactions of each compound with respective binding sites given in Table 3.

**Table 2:** Docking energy values.

	Cnn	Gdp
9a	−9.1	−7.4
9b	−8.9	−7.7
9c	−8.5	−7.3
9d	−7.1	−7.8
9e	−8.8	−8.2
9f	−9.4	−8
9g	−8.7	−8.1
9h	−9.6	−8.3
9i	−8.5	−7.5
9j	9.4	−8
Cn	−7.9	NA
Gdp	NA	−9.4

**Table 3:** Amino acid interactions with the compound in the active site of tubulin.

S. No	Compound	Amino Acid Interaction
1	9FCNN	ASPB:199, LEUB:255, PROB:348, ILEB:347, VALB:257, THRB:314, ASNB:350 – Van der Waals; ASNB:258 – Conventional Hydrogen Bond; ASNB:349 – Carbon Hydrogen Bond; METB:259 – Pi-Sulfur; ALAB:316, ALAB:256, VALB:260, PROB:263, PROB:261 – Pi-Alkyl; LYSB:352 – Alkyl.
2	9HCNN	METB:259, VALB:315, ASNB:350, THRB:314, ASNB:349, VALB:260, VALB:257 – Van der Waals; ASNB:258 – Conventional Hydrogen Bond; PROB:263, ALAB:256, PROB:261, ILEB:347, ALAB:316 – Pi-Alkyl; LEUB:255, LYSB:352 – Alkyl.
3	9JCNN	ASPB:199, VALB:257, VALB:260, ASNB:350, VALB:315, ALAB:316, LEUB:255, LYSB:254, LEUB:248 – Van der Waals; ASNB:258 – Conventional Hydrogen Bond; ASNB:349, THRB:314 – Carbon Hydrogen Bond; ALAB:256, PROB:261, PROB:263, ILEB:347, LYSB:352 – Pi-Alkyl; METB:259 – Alkyl.
4	CNN	THRB:314, ASNB:250, ALAB:354, ALAB:316, VALB:318, ILEB:378, LYSB:254 – Van der Waals; CYSB:241, ASNB:258 – Conventional Hydrogen Bond; LYSB:352 – Carbon Hydrogen Bond; LEUB:255 – Pi-Sigma; META:259 – Pi-Sulfur; LEUB:248, ALAB:250 – Pi-Alkyl; LEUB:242 – Alkyl.
5	9EGDP	ASNB:228, GLYB:225, GLNB:15, THRB:223 MGB:502, SERB:178 – Van der Waals; GLNB:11 – Carbon Hydrogen Bond; ASPB:179 – Pi-Anion; LYSB:19 – Pi-Cation; TYRB:224 – Pi-Pi-shaped; HISB:229 -Pi-Alkyl.
6	9GGDP	VALB:23, ASPB:179, HISB:229, TYRB:224, THRB:223, GLNB:15, GLNB:11, GLUB:71, GYB:73, THRB:74, SERB:77, GLYB:225 – Van der Waals; SERB:232 – Unfavorable Acceptor-Acceptor; LYSB:19 – Pi-Cation.
7	9HGDP	SERB:277, THRB:223, SERB:178, MGB:502, GLNB:11, GLNB:15, GLYB:225, SERB:232 – Van der Waals; LYSB:19 – Conventional Hydrogen Bond; TYRB:224 – Pi-Pi T-Shaped; HISB:229 – Alkyl; ASPB:179 – Pi-Anion.
8	GDP	PROB:173, THRB:180, LYSB:176, METB:398 – Van der Waals; GLNB:394, SERB:174, GLUB:183, SERB:178, VALB:177 – Conventional Hydrogen Bond; PROB:184, PROB:175 – Pi-Alkyl.

## 3 Experimental section

### 3.1 General

All the solvents, catalysts, fine chemicals, salts, and reagents were purchased from AVRA PVT Ltd, Hyderabad. The progress of the organic reactions justified by Merck TLC plates with the utilization of UV light. Both ^1^H & ^13^C spectra were taken from BRUKER NMR (300 MHz, 400 MHz) instrument. Melting points were measured with locally manufactured melting point apparatus.

#### 3.1.1 4-(5-(4,6-Dimorpholino-1,3,5-triazin-2-yl)-1,3,4-oxadiazol-2-yl)phenyl)amine (5)

Compound **3** (5 g, 33.1 mmol) was soluble in POCl_3_ (70 mL) and added 4,6-dimorpholino-1,3,5-triazine-2-carboxylic acid (**4**) (9.7 g, 33.1 mmol). The reaction mass allows for a stir at reflux for 4 h. After the successful of reaction, the solvent was distilled off, and crude was extracted with EtOAc solvent. The extracting mass was washed with aq NaHCO_3_, and the EtOAc layers was separated and dried with Na_2_SO_4_. The pure compound **5** was obtained through column chromatography of crude one with 1:1 EtOAc and hexane solvent mixture. Yield: 5.8 g in 84% yield, as off white solid.^1^H NMR (400 MHz, DMSO‑*d*_6_): *δ* 3.56–3.64 (m, 8H), 3.74–3.82 (m, 8H), 6.11 (brs, 2H), 7.08 (d, 2H, *J* = 7.8 Hz), 7.56 (d, 2H, *J* = 7.8 Hz);^13^C NMR (75 MHz, DMSO‑*d*_6_): *δ* 50.6, 67.4, 115.2, 123.6, 130.4, 149.2, 162.5, 164.5, 165.6, 167.5; MS (ESI): *m*/*z* 411 [M+H]^+^.

#### 3.1.2 2-Azido-N-(4-(5-(4,6-dimorpholino-1,3,5-triazin-2-yl)-1,3,4-oxadiazol-2-yl)phenyl) acetamide (7)

The compound **5** (5.6 g, 13.6 mmol) was soluble in 60 mL of anhydrous dichloro methane. To this, 2-azidoacetic acid (**6**) (1.4 g, 13.6 mmol), EDCI (261 mg, 1.36 mmol) and HOBt (184 mg, 1.36 mmol) reaction mass was added slowly. This setup undergoes stirring at atmospheric temperature over 12 h time period. Now the organic mass washed with sat. NaHCO_3_ solution and extricate with DCM solvent followed by drying over anhyd. Na_2_SO_4_. The pure compound **7** was obtained through column chromatography of crude one with 4:6 EtOAc and hexane solvent mixture. Yield: 5.9 g in 88% yield with off brown solid. Mp: 178–180 °C; ^1^H NMR (400 MHz, DMSO‑*d*_6_): *δ* 3.57–3.65 (m, 8H), 3.75–3.83 (m, 8H), 5.10 (s, 2H), 749 (d, 2H, *J* = 7.3 Hz), 7.67 (d, 2H, *J* = 7.3 Hz); ^13^C NMR (75 MHz, DMSO‑*d*_6_): *δ*50.5, 51.4, 67.3, 118.5, 123.6, 130.4, 138.5, 162.6, 164.2, 165.4, 167.5, 170.4; MS (ESI): *m*/*z* 494 [M+H]^+^.

#### 3.1.3 N-(4-(5-(4,6-Dimorpholino-1,3,5-triazin-2-yl)-1,3,4-oxadiazol-2-yl)phenyl)-2-(4-(3,4,5-trimethoxyphenyl)-1H-1,2,3-triazol-1-yl)acetamide (9a)

The azide **7** (500 mg, 1.01 mmol) and 5-ethynyl-1,2,3-trimethoxybenzene (**8a**) (195 mg, 1.01 mmol) were soluble in 7.5 mL water and 7.5 mL *t*-butyl alcohol. To this 30 mg Sodium ascorbate (15 mol%, 0.151 mmol) and 13 mg copper (II) sulfate pent hydrate (5 mol%, 0.05 mmol) added instantly. This reaction performed under dark conditions over 24 h time period. After successful of the reaction, *tert*-butyl alcohol was distilled off. The obtained aqueous layer extricate with EtOAc trice (each time 30 mL). This organic layer was washed with aqua and dried over sodium sulfate salt followed by distilling the solvent then crude was obtained. The pure compound **9a** was obtained through column chromatography of crude one with 6:4 EtOAc and hexane solvent mixture. Yield: 519.5 mg with 75% with white solid. Mp: 267–269 °C, ^1^H NMR (400 MHz, DMSO‑*d*_6_): *δ* 3.58–3.67 (m, 8H), 3.77–3.92 (brs, 17H), 5.18 (s, 2H), 7.12 (s, 2H), 753 (d, 2H, *J* = 7.4 Hz), 7.69 (d, 2H, *J* = 7.4 Hz), 7.81 (s, 1H);^13^C NMR (75 MHz, DMSO‑*d*_6_): *δ* 50.4, 51.4, 57.5, 57.9, 67.4, 111.6, 118.6, 123.5, 125.3, 130.5, 133.2, 138.4, 140.4, 148.4, 154.2, 162.5, 164.6, 165.3, 167.5, 170.4; MS (ESI): *m*/*z* 686 [M+H]^+^.

#### 3.1.4 2-(4-(3,5-Dimethoxyphenyl)-1H-1,2,3-triazol-1-yl)-N-(4-(5-(4,6-dimorpholino-1,3,5-triazin-2-yl)-1,3,4-oxadiazol-2-yl)phenyl)acetamide (9b)

This derivative **9b** was synthesized through the procedure used for **9a**, employing **7** (500 mg, 1.01 mmol) was soluble in 15 mL of *t*-butyl alcohol:H_2_O, and the addition of 1-ethynyl-3,5-dimethoxybenzene (**8b)** (164 mg, 1.01 mmol) and sodium ascorbate (30 mg, 15 mol%, 0.151 mmol), CuSO_4_·5H_2_O (13 mg, 5 mol%, 0.05 mmol) and pure derivative **9b** was obtained through column chromatography of crude one with 6:4 EtOAc and hexane solvent mixture. Yield: 521.2 mg in 79% yield with white solid. Mp: 279–281 °C, ^1^H NMR (400 MHz, DMSO‑*d*_6_): *δ* 3.53–3.64 (m, 8H), 3.72–3.86 (brs, 8H), 3.90 (s, 6H), 5.23 (s, 2H), 6.54 (s, 1H), 7.04 (s, 2H), 754 (d, 2H, *J* = 7.5 Hz), 7.70 (d, 2H, *J* = 7.5 Hz), 7.83 (s, 1H); ^13^C NMR (75 MHz, DMSO‑*d*_6_): *δ* 50.4, 51.6, 57.4, 67.5, 102.6, 111.6, 118.5, 123.5, 125.4, 130.5133.2, 138.3, 148.4, 161.6, 162.4, 164.3, 165.5, 167.6, 170.5; MS (ESI): *m*/*z* 656 [M+H]^+^.

#### 3.1.5 N-(4-(5-(4,6-Dimorpholino-1,3,5-triazin-2-yl)-1,3,4-oxadiazol-2-yl)phenyl)-2-(4-(4-metho xyphenyl)-1H-1,2,3-triazol-1-yl)acetamide (9c)

This derivative **9c** was synthesized through the procedure used for **9a**, employing **7** (500 mg, 1.01 mmol) was soluble in 15 mL of *t*-butyl alcohol:H_2_O, and the addition of 1-ethynyl-4-methoxybenzene (**8c)** (133 mg, 1.01 mmol) and sodium ascorbate (30 mg, 15 mol%, 0.151 mmol), CuSO_4_·5H_2_O (13 mg, 5 mol%, 0.05 mmol) and pure derivative **9c** was obtained through column chromatography of crude one with 6:4 EtOAc and hexane solvent mixture. Yield: 511.6 mg in 81% with white solid. Mp: 271–273 °C,^1^H NMR (400 MHz, DMSO‑*d*_6_): *δ* 3.53–3.64 (m, 8H), 3.72–3.86 (brs, 8H), 3.89 (s, 3H), 5.25 (s, 2H), 7.15 (s, 2H), 753 (d, 2H, *J* = 7.5 Hz), 7.68 (d, 2H, *J* = 7.5 Hz), 7.84 (s, 1H); ^13^C NMR (75 MHz, DMSO‑*d*_6_): *δ* 50.5, 51.4, 57.5, 67.4, 115.3, 118.5, 123.6, 125.4, 130.2, 130.8, 131.4, 138.5, 148.2, 160.5, 162.6, 164.3, 165.6, 167.5, 170.6; MS (ESI): *m*/*z* 626 [M+H]^+^.

#### 3.1.6 N-(4-(5-(4,6-Dimorpholino-1,3,5-triazin-2-yl)-1,3,4-oxadiazol-2-yl)phenyl)-2-(4-(pyridin-4-yl)-1H-1,2,3-triazol-1-yl)acetamide (9d)

This derivative **9d** was synthesized through the procedure used for **9a**, employing **7** (500 mg, 1.01 mmol) was soluble in 15 mL of *t*-butyl alcohol:H_2_O, and the addition of 4-ethynylpyridine (**8d)** (104 mg, 1.01 mmol) and sodium ascorbate (30 mg, 15 mol%, 0.151 mmol), CuSO_4_·5H_2_O (13 mg, 5 mol%, 0.05 mmol) and pure derivative **9d** was obtained through column chromatography of crude one with 6:4 EtOAc and hexane solvent mixture. Yield: 498.3 mg in 83% with white solid. Mp: 298–300 °C, ^1^H NMR (400 MHz, DMSO‑*d*_6_): *δ* 3.53–3.64 (m, 8H), 3.72–3.86 (brs, 8H), 5.27 (s, 2H), 756 (d, 2H, *J* = 7.6 Hz), 7.71 (d, 2H, *J* = 7.6 Hz), 7.85 (s, 1H), 8.13 (d, 2H, *J* = 8.1 Hz), 8.50 (d, 2H, *J* = 8.1 Hz); ^13^C NMR (75 MHz, DMSO‑*d*_6_): *δ* 50.5, 51.6, 67.4, 118.6, 118.6, 123.5, 125.6, 130.4, 133.5, 138.5, 148.2, 151.2, 162.5, 164.4, 165.5, 167.2, 170.4; MS (ESI): *m*/*z* 596 [M+H]^+^.

#### 3.1.7 2-(4-(4-Chlorophenyl)-1H-1,2,3-triazol-1-yl)-N-(4-(5-(4,6-dimorpholino-1,3,5-triazin-2-yl)-1,3,4-oxadiazol-2-yl)phenyl)acetamide (9e)

This derivative **9e** was synthesized through the procedure used for **9a**, employing **7** (500 mg, 1.01 mmol) was soluble in 15 mL of *t*-butyl alcohol:H_2_O, and the addition of 1-chloro-4-ethynylbenzene (**8e)** (137 mg, 1.01 mmol) and sodium ascorbate (30 mg, 15 mol%, 0.151 mmol), CuSO_4_·5H_2_O (13 mg, 5 mol%, 0.05 mmol) and pure derivative **9e** was obtained through column chromatography of crude one with 6:4 EtOAc and hexane solvent mixture. Yield: 498.3 mg in 80% with white solid. Mp: 276–278 °C, ^1^H NMR (400 MHz, DMSO‑*d*_6_): *δ* 3.53–3.64 (m, 8H), 3.72–3.86 (brs, 8H), 5.32 (s, 2H), 754–7.62 (m, 4H), 7.70–7.78 (m, 4H), 7.87 (s, 1H); ^13^C NMR (75 MHz, DMSO‑*d*_6_): *δ* 50.5, 51.4, 67.4, 118.6, 123.5, 125.3, 129.4, 129.9, 130.2, 130.8, 134.3, 138.4, 148.3, 162.6, 164.5, 165.6, 167.7, 170.6; MS (ESI): *m*/*z* 629 [M − H]^+^

#### 3.1.8 2-(4-(4-Bromophenyl)-1H-1,2,3-triazol-1-yl)-N-(4-(5-(4,6-dimorpholino-1,3,5-triazin-2-yl)-1,3,4-oxadiazol-2-yl)phenyl)acetamide (9f)

This derivative **9f** was synthesized through the procedure used for **9a**, employing **7** (500 mg, 1.01 mmol) was soluble in 15 mL of *t*-butyl alcohol:H_2_O, and the addition of 1-bromo-4-ethynylbenzene (**8f)** (183 mg, 1.01 mmol) and sodium ascorbate (30 mg, 15 mol%, 0.151 mmol), CuSO_4_·5H_2_O (13 mg, 5 mol%, 0.05 mmol) and pure derivative **9f** was obtained through column chromatography of crude one with 6:4 EtOAc and hexane solvent mixture. Yield: 509.3 mg in 74% with off white solid. Mp: 293–295 °C,^1^H NMR (400 MHz, DMSO‑*d*_6_): *δ* 3.54–3.65 (m, 8H), 3.73–3.87 (brs, 8H), 5.35 (s, 2H), 756–7.64 (m, 4H), 7.72–7.80 (m, 4H), 7.88 (s, 1H); ^13^C NMR (75 MHz, DMSO‑*d*_6_): *δ* 50.7, 51.5, 67.4, 118.6, 123.4, 123.8, 125.2, 129.4, 130.5, 130.8, 132.4, 138.4, 148.5, 162.5, 164.4, 165.6, 167.5, 170.6; MS (ESI): *m*/*z* 673 [M − 2]^+^.

#### 3.1.9 N-(4-(5-(4,6-Dimorpholino-1,3,5-triazin-2-yl)-1,3,4-oxadiazol-2-yl)phenyl)-2-(4-(4-nitro phenyl)-1H-1,2,3-triazol-1-yl)acetamide (9g)

This derivative **9g** was synthesized through the procedure used for **9a**, employing **7** (500 mg, 1.01 mmol) was soluble in (15 mL) of *t*-butyl alcohol:H_2_O, and the addition of 1-ethynyl-4-nitrobenzene (**8g)** (148 mg, 1.01 mmol) and sodium ascorbate (30 mg, 15 mol%, 0.151 mmol), CuSO_4_·5H_2_O (13 mg, 5 mol%, 0.05 mmol) and pure derivative **9g** was obtained through column chromatography of crude one with 6:4 EtOAc and hexane solvent mixture. Yield: 525.4 mg in 81% yield with off white solid. Mp: 283–285 °C, ^1^H NMR (400 MHz, DMSO‑*d*_6_): *δ* 3.54–3.65 (m, 8H), 3.73–3.87 (brs, 8H), 5.41 (s, 2H), 7.57 (d, 2H, *J* = 8.2 Hz), 7.74 (d, 2H, *J* = 8.2 Hz), 7.90 (s, 1H), 7.95 (d, 2H, *J* = 7.6 Hz), 8.02 (d, 2H, *J* = 7.6 Hz); ^13^C NMR (75 MHz, DMSO- *d*_*6*_):*δ* 50.5, 51.5, 67.4, 118.6, 121.8, 123.4, 125.3, 128.4, 130.5, 130.6, 138.5, 148.4, 162.5, 164.6, 165.6, 165.4, 167.6, 170.6; MS (ESI): *m*/*z* 641 [M+H]^+^.

#### 3.1.10 2-(4-(3,5-Dimethylphenyl)-1H-1,2,3-triazol-1-yl)-N-(4-(5-(4,6-dimorpholino-1,3,5-triazi n-2-yl)-1,3,4-oxadiazol-2-yl)phenyl)acetamide (9h)

This derivative **9h** was synthesized through the procedure used for **9a**, employing **7** (500 mg, 1.01 mmol) was soluble in (15 mL) of *t*-butyl alcohol:H_2_O, and the addition of 1-ethynyl-3,5-dimethylbenzene (**8h)** (131 mg, 1.01 mmol) and sodium ascorbate (30 mg, 15 mol%, 0.151 mmol), CuSO_4_·5H_2_O (13 mg, 5 mol%, 0.05 mmol) and pure derivative **9h** was obtained through column chromatography of crude one with 6:4 EtOAc and hexane solvent mixture. Yield: 529.4 mg in 84% with off white solid. Mp: 258–260 °C, ^1^H NMR (400 MHz, DMSO‑*d*_6_): *δ* 2.36 (s, 6H), 3.55–3.65 (m, 8H), 3.73–3.87 (brs, 8H), 4.76 (s, 2H), 6.53 (s, 1H), 7.45 (s, 2H), 7.54 (d, 2H, *J* = 7.5 Hz), 7.68 (d, 2H, *J* = 7.5 Hz), 7.80 (s, 1H); ^13^C NMR (75 MHz, DMSO- *d*_*6*_): *δ* 28.5, 50.7, 51.5, 67.4, 118.5, 123.5, 125.4, 128.4, 128.8, 130.2, 133.5, 138.3, 138.9, 148.2, 162.5, 164.4, 165.6, 167.6, 170.6; MS (ESI): *m*/*z* 624 [M+H]^+^.

#### 3.1.11 N-(4-(5-(4,6-Dimorpholino-1,3,5-triazin-2-yl)-1,3,4-oxadiazol-2-yl)phenyl)-2-(4-(p-tolyl)-1H-1,2,3-triazol-1-yl)acetamide (9i)

This derivative **9i** was synthesized through the procedure used for **9a**, employing **7** (500 mg, 1.01 mmol) was soluble in 15 mL of *t*-butyl alcohol:H_2_O, and the addition of 1-ethynyl-4-methylbenzene (**8i)** (117 mg, 1.01 mmol) and sodium ascorbate (30 mg, 15 mol%, 0.151 mmol), CuSO_4_·5H_2_O (13 mg, 5 mol%, 0.05 mmol) and pure derivative **9i** was obtained through column chromatography of crude one with 6:4 EtOAc and hexane solvent mixture. Yield: 488.6 mg in 79% with light yellow solid. Mp: 255–257 °C,^1^H NMR (400 MHz, DMSO‑*d*_6_): *δ* 2.38 (s, 3H), 3.55–3.65 (m, 8H), 3.73–3.87 (brs, 8H), 4.73 (s, 2H), 7.23 (d, 2H, *J* = 7.3 Hz), 7.46 (d, 2H, *J* = 7.3 Hz), 7.55 (d, 2H, *J* = 7.5 Hz), 7.69 (d, 2H, *J* = 7.5 Hz), 7.81 (s, 1H); ^13^C NMR (75 MHz, DMSO‑*d*_6_): *δ* 29.5, 50.6, 51.4, 67.4, 118.6, 123.6, 125.3, 128.2, 130.1, 130.6, 131.4, 138.5, 142.5, 148.3, 162.5, 164.4, 165.5, 167.6, 170.5; MS (ESI): *m*/*z* 610 [M+H]^+^.

#### 3.1.12 N-(4-(5-(4,6-Dimorpholino-1,3,5-triazin-2-yl)-1,3,4-oxadiazol-2-yl)phenyl)-2-(4-(3,5-dinitrophenyl)-1H-1,2,3-triazol-1-yl)acetamide (9j)

This derivative **9j** was synthesized through the procedure used for **9a**, employing **7** (500 mg, 1.01 mmol) was soluble in 15 mL of *t*-butyl alcohol:H_2_O, and the addition of 1-ethynyl-3,5-dinitrobenzene (**8j)** (194 mg, 1.01 mmol) and sodium ascorbate (30 mg, 15 mol%, 0.151 mmol), CuSO_4_·5H_2_O (13 mg, 5 mol%, 0.05 mmol) and pure derivative **9j** was obtained through column chromatography of crude one with 6:4 EtOAc and hexane solvent mixture. Yield: 540.4 mg in 78% off white solid. Mp: 308–310 °C,^1^H NMR (400 MHz, DMSO‑*d*_6_): *δ* 2.38 (s, 3H), 3.55–3.65 (m, 8H), 3.73–3.87 (brs, 8H), 4.73 (s, 2H), 7.58 (d, 2H, *J* = 7.7 Hz), 7.73 (d, 2H, *J* = 7.7 Hz), 7.89 (s, 1H), 8.34 (s, 1H), 8.66 (s, 2H); ^13^C NMR (75 MHz, DMSO- *d*_*6*_): *δ* 50.7, 51.6, 67.5, 116.4, 118.5, 120.4, 123.6, 125.4, 130.4, 133.5, 138.4, 148.3, 162.5, 164.5, 165.6, 165.9, 167.3, 170.6; MS (ESI): *m*/*z* 686 [M+H]^+^.

### 3.2 MTT assay

100 μL medium was employed to inoculate the micro titer 96 well tissue culture plates having 1 × 104 cells. 37 °C temperature was maintained to incubate these plates in 5% CO_2_ humidified incubator over 18 h' time before the starting of experiment. After the removal of old medium, 100 μL fresh medium provided for our test compound derivatives and standard drug at various concentrations like 0.5 μM, 1 μM, and 2 μM respectively. Now it was added to each well and allowed for incubation at 37 °C over 24 h’ time. Now the medium was removed and restore with 10 μL MTT dye. Again, the platers were allowed for incubation at 37 °C over 2h time. The outcoming formazan crystals were dissolved in 100 μL extracted buffer. Optical density (O.D) was recorded at 570 nm with Multi-mode Varioskan Instrument-Themo Scientific micro plate reader. In medium, % of DMSO not exceeded to 0.25%.

### 3.3 Ligand preparation and docking studies

Accelrys Discovery studio version 4.0 was utilized to visualize the ligand structures, receptors, and hydrogen-bonding networks. It was also used to render images. The ligand structures were drawn using the Chemsktech software and were converted to 3 d format saved to mol2 format for further processing. All ligands were Energy minimized by chimera applying 'AMBER’ force field with steepest descent algorithm. Protein was collected from RCSB bank (www.rcsb.org) in PDB ID: 1SA0 (Crystal structures of tubulin complexed with colchicine binding site and GDP binding site, both binding sites selected for this study). The Crystal Structure of the human Tubulin complex with colchicine Protein [1SA0] was resolved using X-ray diffraction method with a resolution factor of 3.58 Å was retrieved from PDB Retrieved structure, which has been further modified for docking calculations. Autodock 4.0 was the primary docking program used for semi-flexible docking studies. Preparation of the ligands and protein receptors in pdbqt file and determination of the grid box size was carried out using Autodock Tools version 1.5.6. A grid box with the dimensions X:30, Y:30, Z:30 Å and a grid spacing of 1.0 Å focused at X: 113, Y:89.289, Z: 7.212 was identified as the protein target colchicine docking site. A grid box with the dimensions X:30, Y:30, Z:30 Å and a grid spacing of 1.0 Å focused at X:97.758 Y:74.4.34 Z: 0.001 was identified as the protein target gdp docking site. The protocol used for performing protein and ligand preparation, along with docking studies, were described elsewhere.

## 4 Conclusion

In summary, a new series of 1,2,3-triazole linked 1,3,4-oxadiazole-triazines (**9a-j**) were designed, synthesized and examined in vitro for their anticancer activity towards PC3 & DU-145, A549 and MCF-7 cancer cell lines by the utilizing of MTT assay with etoposide as standard drug. Among them, compound **9d** with 4-pyridyl moiety showed remarkable anticancer activity (PC3 = 0.17 ± 0.063 μM; A549 = 0.19 ± 0.075 μM; MCF-7 = 0.51 ± 0.083 μM and Du-145 = 0.16 ± 0.083 μM).

## Author contribution statement

Sujana Oggu: Performed the experiments.

Parameswari Akshinthala: Contributed reagents, materials, analysis tools or data.

Naresh Kumar Katari; Srimannarayana Malempati: Conceived and designed the experiments.

Laxmi Kumari Nagarapu; Rambabu Gundla: Analyzed and interpreted the data.

Sreekantha Babu Jonnalagadda: Analyzed and interpreted the data; Wrote the paper.

## Data availability statement

No data was used for the research described in the article.

## Declaration of competing interest

The authors declare that they have no known competing financial interests or personal relationships that could have appeared to influence the work reported in this paper.
